# Introductory Lectures

**Published:** 1848-04

**Authors:** 


INTRODUCTORY LECTURES.

These have been so numerous, that we have found it impossible, without being invidious, to notice any portion of them. We observe it asked in some of our contemporaries, whether the opinions expressed in an Introductory Lecture of a Professor are to be regarded as those of the school to which he is attached? They who are at all familiar with the nature of Introductory Lectures, as generally given, are aware that it is but the opening lecture of the Professor to his particular class; and his expressed opinions are as completely his own on every subject discussed as his private or professional sentiments in general. They never can be regarded as those of the school, unless when distinctly stated by him to be so.

